# Ethical considerations and multidisciplinary care for pediatric patients with hypoplastic left heart syndrome: a narrative review with a systematic search

**DOI:** 10.3389/fcvm.2025.1641337

**Published:** 2025-12-11

**Authors:** Mojca Železnik, Urh Grošelj, Petja Fister

**Affiliations:** 1 Department of Neonatology, University Children’s Hospital, University Medical Centre Ljubljana, Ljubljana, Slovenia; 2 Department of Endocrinology, Diabetes and Metabolism, University Children’s Hospital, University Medical Centre Ljubljana, Ljubljana, Slovenia; 3 Department of Medical Ethics, Faculty of Medicine, University of Ljubljana, Ljubljana, Slovenia; 4 Department of Pediatrics, Faculty of Medicine, University of Ljubljana, Ljubljana, Slovenia; 5 National Medical Ethics Committee, Minister of Health, Ljubljana, Slovenia; 6 Department of Pediatric Critical Care, University Children’s Hospital, University Medical Centre Ljubljana, Ljubljana, Slovenia

**Keywords:** hypoplastic left heart syndrome, congenital heart defect, prenatal diagnosis, neonatal care, palliative surgery, medical ethic, multidisciplinary care, decision making

## Abstract

**Background:**

Hypoplastic left heart syndrome (HLHS) was a fatal congenital heart defect (CHD) until the 1980s. Introduction of the Norwood procedure and subsequent Fontan operation significantly improved survival by creating a single-ventricle circulation. Due to the high mortality associated with the Norwood operation, neonatal orthotopic heart transplantation emerged as an alternative, despite challenges such as lifelong immunosuppressive treatment and uncertain longevity of transplanted hearts.

**Methods:**

A narrative review with a systematic literature search was conducted in the PubMed, following PRISMA guidelines and included studies of ethical and medical considerations, decision-making, counseling and treatment planning in children with HLHS. In addition, we manually screened reference lists to identify further relevant literature. We aimed to explore: how do ethical considerations and decision-making processes influence the management and outcomes of fetuses and neonates with HLHS and their families across prenatal, postnatal, and long-term care?

**Results:**

Of the 115 studies, 56 met the inclusion criteria. Early diagnosis through prenatal fetal ultrasound has markedly improved survival rates by enabling better parental awareness, counseling, and decision-making. Managing HLHS requires urgent, extensive, and costly medical interventions, with outcomes influenced by the healthcare system's expertise, the experience of cardiologists and surgeons, ethical, legal, and religious considerations of the parents and medical team. The prenatal phase is crucial for optimal management, with advanced fetal ultrasound facilitating early detection. Postnatal care involves a multidisciplinary approach, including stage palliation physiology/surgery tailored to each patient. Despite surgical advancements, HLHS patients face higher morbidity and mortality rates than other patients with CHDs, with long-term survival and quality of life remaining key concerns. Ethical considerations play a significant role in the management of HLHS, encompassing the autonomy of families, the best medical interests of the child, societal, and cultural factors. Decision-making must balance full disclosure with sensitivity to parents' values and beliefs.

**Conclusions:**

Management of HLHS involves multidisciplinary approach with complex medical and ethical considerations, but the current literature lacks high-quality studies or consensus guidelines on ethical decision-making. Therefore, the influence of ethical considerations on clinical management and patient care remains unclear, highlighting the need for further research.

## 1 Introduction

Congenital heart defects (CHDs) are the leading cause of children morbidity, occurring in approximately 1 neonate per 100 live births (1–3). Critical CHDs require urgent and extensive medical and surgical care in early childhood to ensure survival (2). In recent decades, treatment and outcomes have improved dramatically; more than 90% of children with repaired CHD survive into adulthood. However, up to 50% of surviving children are at increased risk for a spectrum of neurodevelopmental disorders across the lifespan (4–7).

Hypoplastic left heart syndrome (HLHS) is a severe critical CHD characterized by underdevelopment of the structures of the left side of the heart, including the mitral valve, left ventricle, aortic valve, ascending aorta, and aortic arch (8). Patients present with a wide range of disease severity depending on the degree of outflow obstruction, underdevelopment of left heart structures, intra-atrial connections, and degree of aortic hypoplasia (9, 10). HLHS is generally divided in three morphological subtypes: mitral atresia and aortic atresia, mitral stenosis and aortic atresia, and mitral stenosis and aortic stenosis (11). Risk factors associated for poor outcome include birth weight below 2.5 kg, prematurity (less than 34 weeks of gestation), significant non-cardiac anomalies, presence of genetic or chromosomal anomalies, and various cardiac risk factors—such as an intact or restrictive atrial septum, aortic atresia, mitral atresia, tricuspid regurgitation, poor ventricular function, additional cardiac anomalies, and presentation with shock (12, 13). Based on the presence or absence of these characteristics, patients are stratified into two categories. Those presenting with one or more of the aforementioned risk factors are classified as high-risk, whereas patients without any of these factors are considered standard-risk. Approximately one third of fetuses fall into the high-risk category (14). The prevalence of HLHS varies between 1.9 and 4.6 per 10,000 births and accounts for 1.4% to 3.8% of all CHDs (2, 8).

Before the 1980s, survival was not possible until the Norwood procedure was introduced. Since then, the prognosis for HLHS patients has improved due to advances in prenatal screening, preoperative management, resuscitation, and surgical techniques, as well as the availability of heart transplantation in some centers (15). Nevertheless, morbidity and mortality remain relatively high compared to other CHDs (8). It is important to note that limited information is available regarding long-term survival and the quality of life of adults with HLHS (10, 15).

This narrative review with systematic literature search aims to explore how do ethical considerations and decision-making processes influence the management and outcomes of fetuses and neonates with HLHS and their families across prenatal, postnatal, and long-term care. We seek to provide insights into best practices for prenatal and postnatal management, while addressing the key ethical considerations faced by medical professionals and families.

## 2 Methods

**Figure 1:**
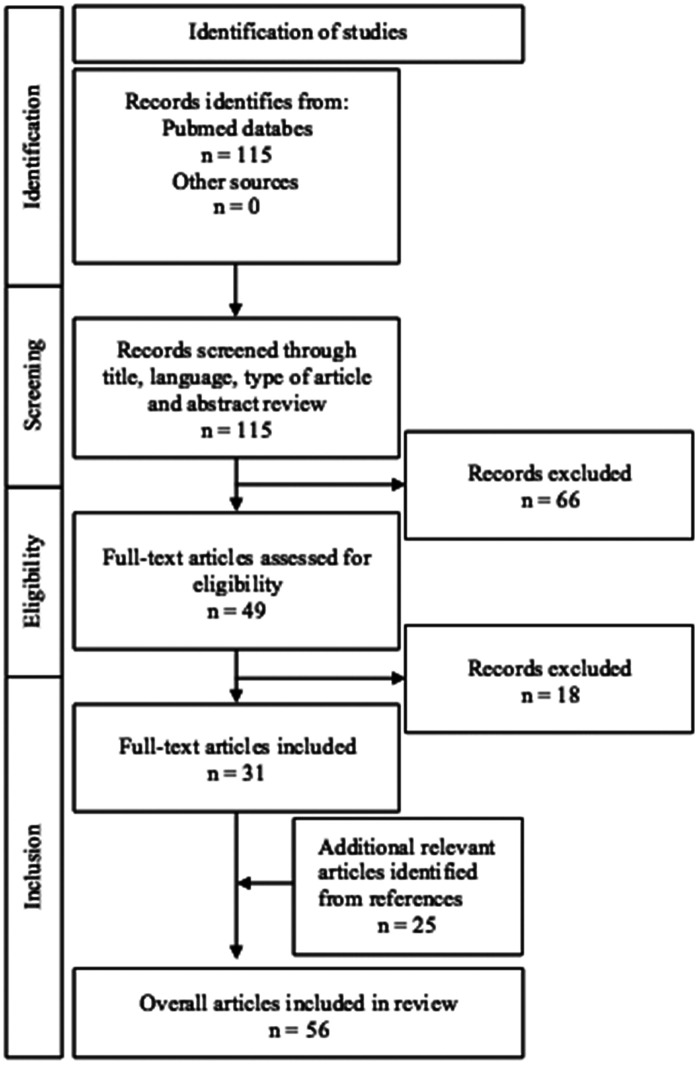
PRISMA flow diagram for a systematic literature search of selection process.

This narrative review with systematic search aimed to synthesize current research on the ethical and medical considerations in management of children with HLHS. We employed the PECO framework to guide our research. PECO is a tool used to structure research questions in evidence-based reviews, standing for Population, Exposure, Comparator, and Outcome. Our research question was: How do ethical considerations and decision-making processes influence the management and outcomes of fetuses and neonates with HLHS, as well as their families, across prenatal, postnatal, and long-term care? We investigated four ethical considerations: autonomy, beneficence, non-maleficence and justice. A comprehensive search was conducted in PubMed database, last updated on April 28, 2025, in accordance with the Preferred Reporting Items for Systematic Reviews and Meta-Analysis (PRISMA) guidelines (Figure 1). Two researchers independently conducted an extensive search of published literature using a pre-defined free-text search strategy. The search string combined keywords related to the disease and ethical considerations, as follows: (“Hypoplastic Left Heart Syndrome” OR “HLHS”) AND (“Ethics” OR “Ethical Issues” OR “Bioethics” OR “Medical Ethics” OR “palliative care” OR “decision making” OR “end-of-life care”). Boolean logic was applied to combine these terms, ensuring a comprehensive retrieval of relevant studies.

Inclusion criteria encompassed English-language articles published between the years 2005 and 2025 that addressed decision-making, management and ethical consideration in HLHS. Exclusion criteria included articles focused primarily on fetal diagnostics, surgical interventions, anesthesia techniques, morphology, physiology, postmortem findings, combined congenital anomalies. Only patients with classic HLHS were included; cases with mixed lesions, such as Shone complex, unbalanced atrioventricular septal defect, borderline left-sided structures, were excluded. Regarding the article type we also excluded case reports, case series, editorials, correspondence, commentaries, or conferences abstracts and those not available at full-text. In addition to database search, we manually screened reference lists to identify further relevant literature.

*Pediatric palliative care* is defined as a specialized medical care focused on relieving suffering and stress in children with serious illnesses. Its primary aim is to enhance the quality of life by addressing physical, emotional, social, and spiritual needs while offering comprehensive support to both the child and their family (16–18). *Pediatric palliative surgery/physiology* refers to surgical and physiological interventions intended to improve function or alleviate symptoms in children with complex or life-limiting congenital conditions. Rather than aiming for a definitive cure, these interventions seek to optimize physiological stability and support survival and development (19). In HLHS, palliative surgery involves a series of staged procedures that are designed to establish a functional circulation and maintain systemic perfusion in the absence of a normal left ventricle. *Ethical decision-making* is defined as the process of exercising professional judgment to make decisions that align with a code of ethics, particularly in contexts where conflicting ethical dimension including autonomy of families, the best medical interests of the child, societal, and cultural factors (20). *Communication with medical teams* refers to any interaction between healthcare professionals and patients or families aimed at sharing information about the patient's condition, prognosis, or treatment options. *Counseling* encompasses structured discussions providing guidance, support, or education to families regarding ethical, emotional, or practical aspects of care (21). *Treatment planning* is a structured, multidisciplinary approach that includes early stabilization (immediate medical interventions to manage symptoms and prepare for surgery), staged surgical interventions (aimed at establishing single-ventricle physiology), and long-term management (ongoing care to monitor and address potential complications and ensure optimal outcomes for the patient) (22).

## 3 Results of systematic literature search

After the selection process of our systematic literature search, we included 31 eligible articled that met our inclusion criteria and followed the review's objectives. We additionally included 25 articles identified from the references (Figure 1).

### 3.1 Prenatal and postnatal phase

#### 3.1.1 Prenatal phase

**Figure 2:**
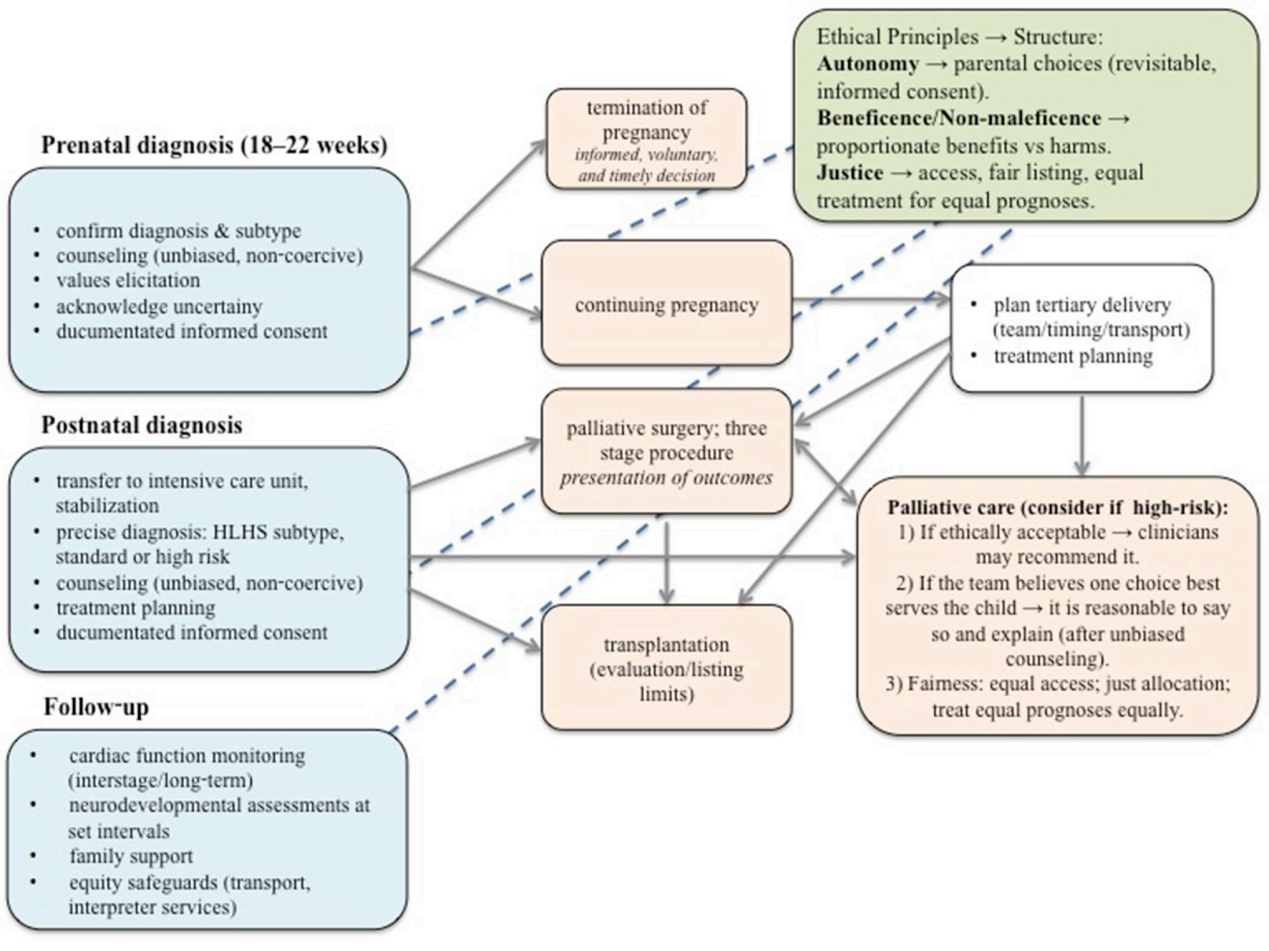
Ethical decision-making pathway for fetuses and neonates with hypoplastic left heart syndrome. The pathway presents a structured approach to ethical decision-making in HLHS, beginning with prenatal diagnosis and extending through postnatal management and long-term follow-up. Blue boxes indicate stages of medical assessment and counseling; orange boxes outline potential clinical pathways, including termination of pregnancy, continuation with palliative surgery, heart transplantation or palliative care. The green box summarizes key ethical principles guiding each decision. Blue lines highlight that ethical considerations are integral throughout all stages of the process, not limited to a single decision point. Decisions in fetus and neonates with HLHS require a balance between medical feasibility, ethical responsibility, and parental values, with continuous reassessment over time as the child's condition evolves. HLHS, hypoplastic left heart syndrome.

With a prenatal diagnosis of HLHS, parents can choose between terminating the pregnancy (an informed, voluntary, and timely decision) or continuing the pregnancy, allowing time to make a treatment plan and prepare for postnatal care. In contrast, a postnatal diagnosis requires parents to make immediate treatment plane for the neonates, providing more concrete information but less time for preparation (Figure 2). The cornerstone of optimal management is reliable prenatal fetal ultrasound diagnosis of CHD. Different countries have different levels of prenatal care, each with its own prenatal CHD detection rate. HLHS can be detected by fetal ultrasound with a four-chamber view of the fetal heart between the 18th and 22nd week of gestation, when the heart structures are well distinguishable, and the fetus is not yet viable (23, 24). Stoll et al. found that fetal echocardiography has a sensitivity of 61.9% for isolated HLHS (25), while a Danish study reported a prenatal detection rate of 92.6% (26). Fetal echocardiography is important for pregnancy counseling, to give the parents the option of pregnancy terminations, and allows to plan delivery in a tertiary care center where specialized surgical treatment is available (23, 27–30). In an Australian survey a prenatal diagnosis was associated with parental choice of surgery (31). However, there can be many obstacles in determining the correct fetal heart anatomy, being the fetal position, the mother's anatomy, and, most importantly, the experience of the ultrasonographer (24, 26). In addition, the patients present with a wide spectrum of disease severity, that cannot be accurately diagnosed and predicted *in utero*, posing an additional challenge for prenatal counseling.

#### 3.1.2 Postnatal phase

For optimal neonatal management, it is best to provide excellent prenatal screening with opportunities for consultation and birth preparation at a tertiary center equipped with an experienced perinatal, neonatal, and anesthesia team. The center should be close to a tertiary cardiac intensive care unit and with short transport routes, as transporting a hemodynamically unstable neonate poses high risks of complications, morbidity and mortality.

Neonates with HLHS are usually born full-term and initially appear to be healthy (9). However, decreased pulmonary vascular resistance, constriction of the ductus arteriosus, and inadequate interatrial connection result in decreased systemic and coronary perfusion, leading to hypoxemia, acidosis, and shock (9, 13). Generally, no heart murmur is detectable or a nonspecific heart murmur is present. Due to aortic atresia, the second heart tone is loud and single. An enlarged liver is palpable due to congestive heart failure (9). After birth, a precise diagnosis of the cardiac and extracardiac structures and a thorough examination of the neonate are required to confirm all prenatal fetal findings and to identify any additional organ malformations. Before the 1980s, there were no treatment options for these neonates; their systemic blood flow was dependent on ductal flow and communication between the left and right atria, and virtually all died in the first week of life (32, 33). Through improvements in cardiac surgery and intensive care, surgical treatment now allows not only survival into infancy, but also life into early adulthood (8).

Neonates with restrictive foramen ovale require atrial septostomy/septectomy after birth (13). In general, surgical treatment of HLHS requires three palliative surgery procedures (Figure 2): The first stage is the Norwood (or Norwood-Sano) procedure or a hybrid procedure for unstable neonates, which is performed within the first two weeks after birth, followed by a Glenn procedure, which is usually performed at 4–6 months of age. The third stage is the Fontan procedure, which is performed at 3–4 years of age (34). Which treatment is chosen depends on the preference and experience of the cardiac surgery team. In the Norwood procedure, an anastomosis is created between the main pulmonary artery and the ascending aortic arch (35). A modified Blalock-Thomas-Taussig shunt is created to supply blood to the lungs, and a connection is made between the right brachiocephalic or subclavian artery and the right pulmonary artery (8, 34). Another alternative is the Sano shunt, this is a connection between the right ventricle and the pulmonary artery (36). The second stage is the Glenn operation, known as the bidirectional cavopulmonary shunt, in which superior vena cava is connected to the cranial part of the right pulmonary artery (34). The third and final stage is the Fontan operation, in which the desaturated systemic venous return from the inferior vena cava is directed through the lateral wall of the right atrium or through the extracardiac conduit into the pulmonary artery (37). While heart transplantation is a treatment option for HLHS, as reported by Kon, only a few studies discuss management starting from the fetal stage. Prenatal diagnosis mainly allows preparation and counseling for postnatal decisions, including palliative surgery, palliative care, or transplantation, but actual transplantation planning is typically made after birth due to limited donor availability and ethical considerations (Figure 2) (38).

A recently published population-based registry reported 5-year survival rate for HLHS born during 1998–2012 in England and Wales was 58.6% and they found that the greatest risk of mortality was associated with the first stage of repair (15). Alternatively to active treatment with palliative surgeries, another option is limitation of active treatment and palliative care, which was the only option available before the Norwood procedure. In a series of 240 fetuses from the Children's Hospital of Philadelphia, 11% opted for termination of pregnancy, while 7% opted for palliative care after birth, in equal proportion of standard or high-risk patients (14, 39). In 11 cases, all of which were high-risk, the family initially favored intensive neonatal care at prenatal consultation, but after birth, it was found that the neonate's condition was severely compromised, and support was withdrawn, or the neonate died before surgery could be performed (39). The lack of intervention should be discussed with families at the time of initial prenatal or postnatal diagnosis (9, 30).

### 3.2 Ethical and communicational aspects

#### 3.2.1 Supporting the autonomy of the family

Prenatal counseling for CHD should include the key content elements, but it is not standardized among centers (14). It can be conveyed to parents by different specialists: obstetricians, perinatologists, pediatric cardiologists, or pediatric cardiac surgeons (30, 40, 41). It is understandable that each specialists has their own physician bias, which can be difficult to understand or even confusing for some of the parents, making clear communication with medical team crucial (29, 40, 42–44).

The information provided to the family should include a clear description of the disease, the medical and surgical treatment possibilities with its complications and burdens, and the long-term prognosis for each option, alongside with mentioning the blind spots in anticipation because of new and evolving management technologies (45, 46). The information should be conveyed in an understandable language, tailored to each of the parent based on their intelligence, education, character, past experiences, their values, beliefs, health care needs, and affordability (40). The informed consent must be unbiased and noncoercive (47, 48). There must be a balance between “ethical obligation for full disclosure” and the doctor “sensitivity to the parents” cultural, educational, religious, and life values (40, 49). However, it is very hard to presume whether the parents have heard all the facts about their child's CHD, or the information was accepted only partially, usually only the positive data believing their child is a fighter and he can take it all and survive. Another very important aspect of sharing information with parent of a fetus or neonate with HLHS is that there is a huge discrepancy in different clinical communities about the best treatment of the disease in the broadest sense possible. Options range from termination of pregnancy, palliative care after the neonate's birth, to offering a series of at least three palliative surgeries before or taking the child directly to cardiac transplantation list, with the concern of never getting a suitable organ (28, 29, 50–52). As one source notes, “Currently there is enormous variation in what parents are told about HLHS.” (47).

Additionally, every CHD and its hemodynamics is literally unique for the child and it has its fetal and neonatal evolution, no two children with CHD have exactly the same condition. An important point to convey to the parents of a child with a complex CHD is the fact that no intervention or surgical procedure can restore to normal what nature has embryologically developed differently. Except in the case of heart transplant, the child will never have a normal, healthy heart. It is for the parents and the broader family to understand that the child will be a chronic cardiac patient and should be regarded as such in all circumstances. When following up children born with HLHS and their families the advice “Treat your child normally” turned out to suggest that the use of normalization by the parents prevented them to seek early interventions when they acknowledged their child's developmental delays (53).

The information shared should include the surgical outcomes at their respective center, other centers and description of complications of the palliative surgeries that the child will need (40). It is crucial to consider the risk of neurodevelopmental delays associated with the procedures, postoperative therapy, expected longevity, and the potential need for a heart transplant, along with its complications and pitfalls (44). Furthermore, it is impossible to predict the specific complications any given neonate may experience in the near or distant future (7, 40). The information should cover the most unfavorable case as well as all situations in between (Figure 2) (54, 55).

#### 3.2.2 Determining the best medical interest of the child—beneficence

For children born with HLHS, beneficence involves optimizing of their physical, emotional, and social outcomes. This requires comprehensive consecutive assessments, communication with medical teams, all while considering the family's preferences and values (56).

As previously noted, no child with complex CHD has the same condition or malformation. Consequently, the management and plan should be tailored to each individual personally. It is advised that in the case of anomalies with an extremely poor outcome approach to counseling may be more directive (14).

Neonatal circulation and hemodynamics need some time after birth to transition from fetal to adult conditions and a subtle and gentle stabilization of neonate's vital functions is preferred with at least possible and noninvasive interventions, which offer safe conditions for the neonate. Once initial stabilization is achieved, a multidisciplinary medical team meeting should be held to review all the data and determine the best course treatment. The decision is later conveyed to parents by the members of the multidisciplinary medical teams, there are different recommendations on who should be included, at least neonatologists, pediatric intensivists, pediatric cardiologists, pediatric cardiac surgeons, intervention cardiologists, anesthesiologists and doctors from pediatric palliative team (57–59).

As mentioned in the previous section, issues about the informed consent, bioethical equipoise, and physician's bias arise once again in the ethical decision-making process. It was stated that even that the experience of a certain specialist have unequal weights in the eyes and ears of the parents (40, 60). In surveys were doctors were asked to imagine their own infant diagnosed with HLHS, their choices were not associated with their estimates of surgical outcomes: even when predicting more than 90% rate for 1 year surgical survival, half of them would choose palliative care or were uncertain about which option they would choose. When comparing the responses of congenital cardiac surgeons in 1999 and 2007, a nonsignificant trend away from palliative surgery was noted, despite improvements in surgical outcomes (54). A similar message was noticed in a survey from Norwegian parents of neonates born with HLHS, those who have chosen palliative care for their child were more educated and more likely associated with the healthcare profession (61). These trends mirror findings from another study, where approximately half of pediatric residents and nurses would choice for pregnancy termination or decline palliative surgery if their own fetus or newborn had HLHS, largely due to concerns about long-term quality of life and survival (62). Furthermore, a recent Swedish cohort study found a significant increase in terminations of pregnancy, rising from 19% in 1993–2000 to 56% in 2001–2010 and consequently a decrease in the incidence of neonates with HLHS, offset by a higher proportion of infants palliative surgery (63).

This highlights that decisions around treatment outcomes are influenced not only by individual specialists' experiences but also by the specific institution in which the care is provided, not to forget the counseling pattern, which impact parental perceptions and decisions (Figure 2) (51, 52).

#### 3.2.3 Avoiding potential harms—non-maleficence

Non-maleficence—not harming the patient, includes vigilance about potential harms associated with treatments and interventions. In the management of children with HLHS, it is advisable constantly assessing benefits and risks by carefully weighing the potential benefits of procedures and surgeries against the associated risks, such as complications from anesthesia or post-operative complications, including the length of mechanical ventilation, hospital stay, and neurodevelopment concerns. The child should be continuously followed at regular check-ups to find any adverse effects from procedures or medications, while doctors are vigilant to tailor the treatment as tolerated for the patient. When curative management is not feasible, supportive care should be offered to ensure the best quality of life for the child and their family.

All the time the neurodevelopment of the child with HLHS is at risk: the brain may be abnormally developed *in utero*, and all the surgical procedures and treatment in intensive care unit bring risks of neurological damage. Recent statements by American Heart Association's highlights the importance of early evaluations for developmental delays at key developmental milestones—6 months, 18 months, 3 years, and 5 years. These assessments are crucial for timely interventions and improving long-term outcomes for child with CHD (6). A great proportion of infants suffer neurodevelopmental delays, limitations of physical activity and later in life limitations in self-care and independent living (7, 64). It is now well-established that neonates with HLHS can survive after a series of palliative surgeries and prolonged intensive care, but this comes with a high risk of physical and neurodevelopmental delays (7, 40). Life seems to be a long struggle for neonates born with HLHS, difficult and painful for the child and emotionally difficult for the parents (65).

After neonate recovers from the first surgical procedure and is transferred from intensive care unit to cardiac unit, the parents are educated on caring for the child, administering medications, and recognizing normal behaviors, so they can safely return home. Regular check-ups are planned and special precautions taken to prevent deteriorations, personally tailored vaccination program is prescribed. Taking into account that greatest risk of mortality is associated with and after the first stage of palliative surgery (Figure 2) (15).

The first palliative surgery is followed by second and third one. Again extensive and aggressive medical and surgical care is needed which to the child bring a certain extent of pain and suffering. Palliative physiology, such as Fontan circulation is not a normal physiological state; it is a hemodynamically unique state not present alone in nature with a lot of special considerations, such as exercise tolerance, atrial arrhythmias, protein-losing entheropathy, and plastic bronchitis. Multiple and prolonged admissions to the hospital are frightening and painful for the child, and have negative physical and emotional risks (38).

#### 3.2.4 Justice with societal and cultural aspects

In healthcare, justice refers to the fair distribution of resources and equal access to care. All the children born with HLHS, regardless of socioeconomic status, geographical location, or background, should have access to necessary diagnostics, treatment, and follow-up care. However, multiple access barriers can affect both counseling and outcome, including geography (country, geography, travel distance and time to specialized centers), type of insurance or coverage, language and cultural differences, and the experience of tertiary center. Furthermore, in situations with limited resources, such as fetal diagnostics, medical transport, staged palliation, transplant accessibility, home monitoring, medical and neurodevelopmental follow-up also define the medical management of patients with HLHS. In situations with limited resources, there should be fair resource allocation for the expensive advanced management. Doctors, nurses, and other healthcare providers should be advocating for policies that support equitable healthcare support for all children with complex diagnoses (66).

The palliative surgeries on one hand and heart transplantation on the other hand that the child with HLHS needs are very expensive for society. Reports from emerging economies highlight the challenges of treating HLHS (67, 68). Even in high-resource settings, access or insurance barriers may delay interventions or limit parenteral counseling options. These disparities underscore the importance of targeted policies and advocacy to support equitable healthcare access. Despite growing awareness of access disparities, evidence quantifying the impact of geographic, socioeconomic, and cultural barriers on HLHS outcomes remains limited. Future studies should consider measurable equity metrics, such as time to first intervention, access to specialized centers, enrollment in follow-up programs, and patient/caregiver satisfaction, to systematically evaluate and address these disparities. By explicitly identifying barriers and their impact, and proposing mitigation strategies, healthcare teams can improve counseling, ethical decision-making, and long-term outcomes for children with HLHS across diverse settings. Additionally, there is debate about offering complex surgical procedures to foreign patients in countries where local healthcare systems are already operating at full capacity. This raises ethical concerns about the allocation of resources and the treatment of foreign patients when domestic needs are urgent.

#### 3.2.5 Active vs. palliative care

In the aspect of appropriateness of offering only palliative care, Mercurio et al. suggest three guidelines to locate the threshold of acceptability. First: if the choice of offering only palliative care is ethically acceptable, we may advise it. Second: when we feel strongly that parents should decide in a certain way, it would be reasonable of us to say so and provide the reasons after the relevant information with all options is presented in unbiased and non-coercive manner. Third: offering only palliative care should be fair in the sense of access to care, fair allocation of resources and maybe most importantly, treating those with equal prognoses equally (Figure 2) (47).

In some cultures, palliative care is not acceptable because parents may feel guilty about not actively attempting to save their child's life (67, 69). It is unreasonable to expect parents to make a decision to withhold treatment, especially when facing a disease or anomalies with an extremely poor prognosis. In such cases, more directive counseling may be necessary (14).

The question posed by Rychik (14): “Whether we have approached the threshold for possibly excluding the discussion of palliative care for uncomplicated HLHS is uncertain”, especially in low income and not well-developed countries, in places where pediatric surgery has not full resources or credentials. Is it fair of foreign centers to take care of foreign patients, possibly at the expense of their own? In general, pediatrician's mission and full responsibility is to protect and promote the best interest of the child, which in the case of a child being born with HLHS is entwined with the interests of close others, social and financial constraints and never knowing what complications and novelties the future is going to bring (70). Since the new therapies are constantly developing, the outcome data should be updated and refined so as our counseling should be subjected to constant review.

#### 3.2.6 Future directions: emerging technologies

Given rapid advances in emerging technologies, particularly in medicine and artificial intelligence (AI), the latter may have a potential role in prenatal echocardiography, risk stratification and perioperative planning with 3D modeling and also long-term neurodevelopmental prognostication. In the future we might suggest a model validation from multi-center cohorts, incorporating diverse anatomic, social and cultural factors into shared ethical decision-making and the best interest of the child. AI tools could help foresee possible choices and outcomes for both the child and the family.

But on the other hand there are so many objective unknowns in the diagnostic and management pathways that fetuses, neonates and children with HLHS and their family face. As mentioned, determining the exact anatomy of the fetal heart and other organs anatomy introduces uncertainty for future prognostication. Postnatally, ongoing diagnostics is continued, procedures and potential complications may generate new data that can change the prognostic results. During the AI-assisted processes, the human intelligence remains essential to supervise investigations and procedures in to maintain communication with the family.

Future management of HLHS may include a combination of approaches, which could include clinical strategies (e.g., multidisciplinary care models, prenatal counseling protocols), ethical frameworks (e.g., shared ethical decision-making, cultural sensitivity in end-of-life discussions), or policy-level recommendations (e.g., guidelines for equitable access to surgery or palliative care).

## 3.3 Discussion

Caring for pediatric patients with HLHS is a complex and multifaceted challenge that requires a multidisciplinary approach. Advances in surgical techniques, such as the Norwood and Fontan procedures, and improvements in prenatal diagnosis have significantly enhanced survival rates. However, these interventions come with high costs and substantial medical and ethical considerations. Ethical considerations may evolve alongside improvements in surgical outcomes and changes in clinical strategies, as better survival and neurodevelopmental outcomes after active care may lead parents to favor active over palliative care. Multidisciplinary approaches enable more precise and thorough counseling, which can further influence parental decision-making and ethical considerations. While surgical advances improve outcomes, they do not eliminate ethical considerations; rather, they shift the balance of risks and benefits, thereby shaping the information and guidance provided to families and influencing their choices.

The role of prenatal counseling is crucial, yet it remains inconsistent across different centers and specialists. Clear, unbiased, and comprehensive communication tailored to the parents' backgrounds is essential to support informed decision-making. Each CHD case is unique, necessitating personalized treatment plans and continuous evaluation.

The ethical issues associated with HLHS management, including decisions about aggressive treatment vs. palliative care, are profound. These decisions are influenced by cultural, societal, and individual values, requiring sensitive and directive counseling when necessary.

Despite medical advancements, children with HLHS often face long-term physical and neurodevelopmental challenges, and their care imposes significant emotional and financial burdens on families and healthcare systems. Therefore, ongoing research, ethical reflection, and support for affected families are critical to improving outcomes and quality of life for these patients.

A key limitation of our study is its methodology as a narrative review with a systematic search relying solely on one database, which may impact the comprehensiveness and rigor. Additionally, we did not use MeSH terms, which may have reduced search sensitivity. We also focused exclusively on classic HLHS, without analyzing subtypes, mixed lesion, or fetal “borderline left ventricles” cohorts separately. Unlike a full systematic review, we did not perform formal quality appraisal of included studies or synthesize data quantitatively, which limits the ability to provide high-level evidence. Most importantly, there is a lack of high-quality, comprehensive studies and consensus-based guidelines that specifically address ethical considerations and decision-making in HLHS management, which limits the ability to draw strong, evidence-supported conclusions. Future studies could address these limitations by conducting a comprehensive systematic review across multiple databases, formally assessing study quality, and targeted investigations of ethical decision-making to provide higher-level evidence and more robust conclusions.

In conclusion, the management of HLHS underscores the importance of a multidisciplinary approach that integrates medical, ethical, and psychosocial considerations to provide the best possible care for these vulnerable children and their families. While ethical principles such as autonomy, beneficence, non-maleficence, and justice play a central role in counseling and decision-making, the current literature provides only partial insight into how these principles are applied in everyday practice. Our review reflects the best available evidence, while highlighting the need for further research and development of structured, consensus-driven guidance to support clinicians and families in navigating these complex decisions.

## Data Availability

The original contributions presented in the study are included in the article/Supplementary Material, further inquiries can be directed to the corresponding author.
